# Integration of GPS, Monocular Vision, and High Definition (HD) Map for Accurate Vehicle Localization

**DOI:** 10.3390/s18103270

**Published:** 2018-09-28

**Authors:** Hao Cai, Zhaozheng Hu, Gang Huang, Dunyao Zhu, Xiaocong Su

**Affiliations:** 1 School of Computer Science and Technology, Wuhan University of Technology, Wuhan 430063, China; caihao@whut.edu.cn; 2 ITS Research Center, Wuhan University of Technology, Wuhan 430063, China; hg@whut.edu.cn (G.H.); 207026@whut.edu.cn (D.Z.); 3 Kotei Technology Company, Wuhan 430200, China; xiaocongs@kotei-info.com

**Keywords:** vehicle localization, High Definition (HD) map, monocular vision, GPS, Kalman filter, sensor fusion

## Abstract

Self-localization is a crucial task for intelligent vehicles. Existing localization methods usually require high-cost IMU (Inertial Measurement Unit) or expensive LiDAR sensors (e.g., Velodyne HDL-64E). In this paper, we propose a low-cost yet accurate localization solution by using a custom-level GPS receiver and a low-cost camera with the support of HD map. Unlike existing HD map-based methods, which usually requires unique landmarks within the sensed range, the proposed method utilizes common lane lines for vehicle localization by using Kalman filter to fuse the GPS, monocular vision, and HD map for more accurate vehicle localization. In the Kalman filter framework, the observations consist of two parts. One is the raw GPS coordinate. The other is the lateral distance between the vehicle and the lane, which is computed from the monocular camera. The HD map plays the role of providing reference position information and correlating the local lateral distance from the vision and the GPS coordinates so as to formulate a linear Kalman filter. In the prediction step, we propose using a data-driven motion model rather than a Kinematic model, which is more adaptive and flexible. The proposed method has been tested with both simulation data and real data collected in the field. The results demonstrate that the localization errors from the proposed method are less than half or even one-third of the original GPS positioning errors by using low cost sensors with HD map support. Experimental results also demonstrate that the integration of the proposed method into existing ones can greatly enhance the localization results.

## 1. Introduction

Intelligent vehicles are emerging technologies that have great potential to enhance driving safety and improve transportation efficiency [1]. A typical intelligent vehicle consists of the modules of perception, decision-making, path planning, and control. Vehicle localization, which is the process of determining the positions and poses of the vehicle, is a step that is crucial for all these modules [2,3]. 

Generally, existing methods of vehicle localization can be classified into three categories. (i) The first one is the Global Navigation Satellite System (GNSS) related methods, such as GPS, Beidou, etc. As raw GPS receivers only provide low positioning accuracy (e.g., as low as 10-m accuracy or less), it is not feasible for intelligent vehicle applications. Usually, GPS is integrated with other sensors (i.e., Inertial Measurement Unit (IMU) [4], Real-time Kinematic (RTK), etc.) for more accurate accuracy. For example, the integration of GPS and IMU can yield less than 10 cm positioning errors [5]. The Differential GPS (D-GPS) methods can also reach high position accuracy (i.e., 5 cm or less) based on the base stations and one popular approach is the RTK methods [6,7]. However, these methods are usually very costly in practice. Moreover, they all suffer from the GPS-blind problems, especially in urban environments [8]. (ii) The second category is for the vision-based methods, which are based on the low-cost visual sensors [9]. Visual sensors have the ability to acquire large amounts of information with only one snapshot. They can perform as the main sensor, where no GPS signals are used [10]. Gakne developed an image-based multipath mitigation approach method when the GNSS signals are subject to severe multipath in urban canyons [11]. One popular method is to use a monocular camera for lane detection, therefore to compute the relative position between the camera and the lanes [12]. However, only traverse position is computed for vehicle self-localization. Visual Odometry (VO) or Simultaneous Localization and Mapping (SLAM) methods are also investigated for vehicle self-localization [13,14]. However, Visual Odometry has serious drift error problem, which is vital for open road scenarios. David et al. present a robust visual localization technique based on an omnidirectional SLAM approach [15]. Raúl et al. present a feature-based monocular simultaneous localization and mapping system [16]. It can run very fast to achieve real-time effect. But vision SLAM methods suffer from the poor robustness in front-end loop closure detection and the complicated after-end optimization [17]. Recently, the role of HD Maps is becoming more and more important for intelligent vehicles [18]. It is believed that HD map is an indispensable part in autonomous driving [19]. HD maps are already available in many countries. Such as the navigation technology company TomTom announced the total coverage of TomTom HD maps globally now is nearly 380,000 km (236,121 miles) [20]. So, the HD map can be as a wide-open approach (just like the daily used map) in the field of intelligent vehicles. In the recent research, these maps have extremely high precision at centimeter-level that are particularly built for self-driving purposes [21]. Many researchers demonstrate that HD map can enhance vision-based vehicle localization. The principle is to correlate image data to the information from the map to improve localization results. For example, Markus et al. use a highly accurate map and a stereo camera for vehicle localization [22]. In the map, all the lane lines and markings are accurately coordinated to providing reference positions. Tao et al. [23] get the position information of landmark first. Then, the resulting map allows for the exploitation of camera-detected features for autonomous real-time localization. Most people do the work of map-matching; it has a high demand for the map and a certain degree of difficulty in landmark detection. To create such a map is time-consuming to build and need to be upgraded regularly. However, we should have a simpler requirement, as if we just only need the line segment equation, not the coordinates of every point. Something should also be done after matching for the localization. And some methods try to integrate the pavement marking and HD map for vehicle positioning [24,25]. However, as pavement markings are sparely distributed and high repetitive, these methods are not practical enough in the field. (iii) The last category is for the LiDAR based localization methods [26,27]. One state-of-the-art method was proposed by Apollo team by using a Velodyne HDL-64E. They used LiDAR data for map generation and localized the vehicle by matching LiDAR data in cell-grid scale with 5–10 cm accuracy. However, such method relies on the high-cost laser hardware (i.e., Velodyne HDL-64E). High-accuracy localization methods usually require high-cost devices, such as sufficient number of RTK stations, IMU, and high definition LiDAR, etc. It is important to develop low-cost solution to localization by fusing different sensor types. And laser SLAM is also investigated for vehicle localization. Basically, high-accuracy vehicle localization usually requires high-cost devices, such as IMU, high definition laser, and sufficient number of differential work stations, etc. 

In this paper, we proposed a new localization method for intelligent vehicles by integrating GPS, monocular vision, and HD map; with a low-cost monocular camera, a common GPS, and lane-level HD map. The proposed method requires no high-cost devices, such as IMU or high definition Laser, and suggests a low-cost yet accurate solution to intelligent vehicle localization. In our method, the raw GPS and vision data are fused within a Kalman framework with lane-level HD map support. In our former works, we proposed a pre-built map-based vision localization method in [28]. It doesn’t use any road landmarks. We tried to code pavement lanes in different colors such that we can derive unique lane marking within sensed range for vehicle localization in [29]. Hence, coding is the main contribution in [29]. However, in this paper, we don’t need unique landmarks. Instead, we can utilize common lane lines for vehicle localization. In addition, the proposed method can be integrated into existing localization methods to enhance localization accuracy. The main contributions of this paper, in contrast to the previous work, are summarized as follows:

(1) We propose using linear Kalman filter to fuse GPS, monocular vision, and HD map and developed a low-cost and accurate solution to vehicle localization;
(2) We propose using HD map to provide reference positions so as to correlate the GPS coordinates and lateral distance from monocular vision, which finally formulates two types of observations or measurements for the developed linear Kalman filter. The HD maps allow the integration of local measurements (i.e., lateral distances from camera) and global measurements into a linear Kalman framework. As a result, we can utilize common lane lines for localization, compared to the unique landmark requirements in existing HD map-based methods;
(3) We develop a data-driven motion model rather than Kinematics one for state prediction and transition for the Kalman filter. The data-driven model is trained from vehicle’s historic trajectories and requires no acceleration or velocity inputs, which therefore requires no high-cost IMU. In addition, it is more flexible and adaptive than the existing Kinematic model used in the literature.

## 2. The Proposed Methods

**Figure 1:**
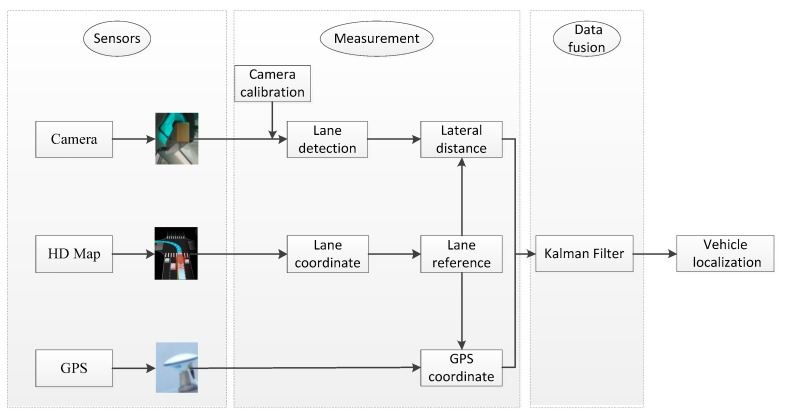
Flowchart of the proposed method.

The flowchart of the proposed method is illustrated in Figure 1. Three types of sensors, i.e., GPS receiver, monocular camera and HD map (HD map is also regarded as map sensor in the field of intelligent vehicles), generate the image, GPS, position reference data, respectively. An input image, captured by the vehicle-borne monocular camera, is first processed to compute the lateral distance between the vehicle and the nearby lanes from camera calibration results. In the meanwhile, we also collect the GPS coordinates from a custom-level GPS receiver. By referring to the HD map, we can correlate the lateral distance from monocular vision and GPS coordinates, and feed them as the measurements into a Kalman filter. As a result, an improved vehicle localization result is derived from the Kalman filter output. Note that the monocular camera and the GPS receiver should be precisely synchronized and calibrated in order for fusion. 

### 2.1. GPS and HD Map

In this proposal, we use a custom-level GPS receiver to collect raw GPS data. For geometry computation purpose, we transform all the original GPS coordinates into a Cartesian coordinate system. In this paper, all the GPS coordinates are transformed into the Universal Transverse Mercator (UTM) coordinate system.

**Figure 2:**
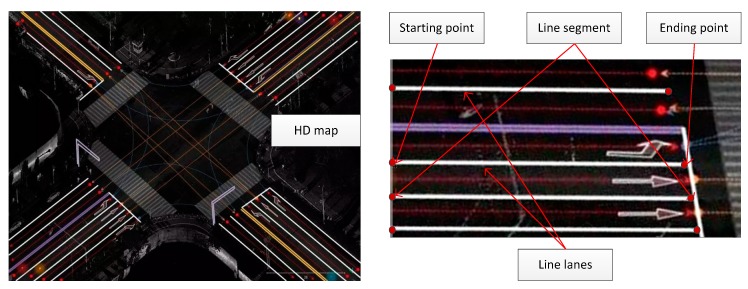
HD map demonstration and the representation of lane line with line segments.

Although the importance of HD map is widely recognized for intelligent vehicles, the definitions of HD map are different from countries to countries. Actually, many map providers, such as Google, Uber, Zenrin, TomTom, NDS, etc.; have their own definitions for HD map. In this paper, we used a simplified lane-level HD map for vehicle localization. In this map, all the lanes and pavement markings are well coordinated with high-precision GPS coordinates (e.g., 3 cm accuracy). Figure 2 demonstrate a HD map produced by Kotei Technology Company (a map company located in Wuhan City), and the representation of lane lines with high-precision GPS coordinates. 

As shown in Figure 2, lane lines are represented with line segments in the HD map. And each line segment consists of two points including the starting and the ending points. As a result, we can quickly compute the corresponding line equation in the UTM system from it. 

The line can also be expressed by using a unit direction and a distance to the origin from Equation (1) as follow.

(1) $$n_{xi}x^{x}+n_{yi}x^{y}+d_{i}=0\;$$

Note that we also need to transform all the GPS coordinates in HD map into the UTM system before we compute line equations with Equations (2) and (3) as follows [23].

(2) $$x=K\ln[tg(\frac{\pi }{4}+\frac{B}{2})*{\left(\frac{1-𝓁\sin B}{1+𝓁\sin B}\right)}^{\frac{𝓁}{2}}]\;$$

(3) $$y=K(L-L_{o})\;$$

where *B* and *L* are the latitude and longitude accepted by GPS, *L*_0_ is the Origin Latitude. *x* and *y* are the result in the UTM coordinate system. *K* can be computed by the information of *B* and *L*, and more details for such transformation is referred to [30].

### 2.2. Lateral Distance Computation from Monocular Vision

In this part, we focus on real-time computation of the distances of the vehicle to lane lines. The calculation of lateral distance is divided into 2 steps: lane detection and camera calibration. The lane detection algorithm is very mature and has been applied in industry. In this paper, we used the method in [31]. 

From the detected lane lines, we can use algorithms to automatically localize a vehicle from camera calibration results. The calibration of an in-vehicle monocular camera consists of intrinsic calibration, as well as extrinsic calibration, a step to compute the pavement geometry in the camera coordinate system. 

The intrinsic parameters of the camera can be calibrated by using the well-known chessboard plane-based method. We propose a novel method to pavement-camera geometry. Unlike the method used in the literature, the proposed method uses vanishing line instead of vanishing point for accurate camera pavement geometry calibration. Most previous studies assume that the x-axis of the camera (the camera coordinate system) is parallel to the traverse direction of the pavement so that the vanishing line of the pavement plane is parallel to x-axis of the image coordinate system. However, this assumption is not true in reality since there may be angles between the x-axis of the camera and the pavement traverse direction. Therefore, a vanishing line based model should be applied. The vanishing line can be determined from at least two vanishing points of different directions on the pavement plane. Note that the camera has fixed relative position with the pavement. Hence, the vanishing points can be computed from multiple video log images. As a result, the vanishing line can be fitted by using Least Mean Square Error (LMSE) method. The camera is calibrated in advance with Zhang’s method [32]. With a calibrated camera and the vanishing line, the pavement plane normal is computed as:

(4) $$n_{pav}=K^{T}l_{pav}\;$$

From the computed pavement normal direction, we can compute the homography between the pavement plane and its image by

(5) $$H=[Kn_{1}\;Kn_{2}\;\lambda g]\;$$

where *g* is the image of the origin of the pavement coordinate system and *λ* is a scale, which can be determined from a reference length (e.g., lane width). The two directions of $n_{1}$ and $n_{2}$ satisfy the following condition:

(6) $$\{\begin{array}{c} n_{1}^{T}n_{pav}=0\\ n_{2}^{T}n_{pav}=0\\ n_{2}^{T}n_{1}=0\\ \left\|n_{1}\right\|=\left\|n_{2}\right\|=1 \end{array}\;$$

Obviously, the choice of $n_{1}$ and $n_{2}$ is not unique from Equation (6). $n_{1}$ and $n_{2}$ can be set from the pavement configuration. For example, the driving direction (lane direction) is set as $n_{1}$ and the traverse direction as $n_{2}$. Note that the image *g*, also the image of the origin of the pavement coordinate system, is not in the camera coordinate system. From Equation (7), we can derive the rotation R and the translation t as

(7) $$\left[\begin{array}{cc} R & t \end{array}\right]=[\begin{array}{cccc} n_{1} & n_{2} & n_{pav} & \lambda K^{-1}g \end{array}]\;$$

With the computation rotation and translation, we can derive the lane equation and the camera focal point coordinates in the world coordinate system by

(8) $$l≅HL_{w}\to L_{w}≅H^{-1}l\;$$

(9) $$O_{c}≅RO_{w}+t\to O_{w}≅R^{-1}O_{c}-R^{-1}t\;$$

where l is the lane in the image coordinate system, $L_{w}$ is the lane in the world coordinate system, Oc is the camera optical center with the correspondence $O_{w}$ in the world coordinate system. Hence, we can compute the lateral distance from the orthogonal projection of the camera (with $O_{W}^{Z}=0$) to the lanes as follows

(10) $$d=L_{w}{}^{T}\left[\begin{array}{ccc} o_{W}^{x} & o_{W}^{y} & 1 \end{array}\right]\;$$

### 2.3. Kalman Filter for GPS, Vision and HD Map Fusion

In this paper, we utilized Kalman filter to fuse GPS, monocular vision, and HD map to enhance vehicle localization. Kalman filtering, also known as linear quadratic estimation (LQE), is an optimal recursive data processing algorithm [33,34]. A typical Kalman filter consists of two steps, the prediction step and the update step. The predict phase is also called priori state estimate. It uses the previous state to compute the current one. The update phase is a posteriori state estimate by fusing the observations and the predicted state from the prediction step. In this paper, we developed a data-driven motion model, compared to Kinematic ones in existing localization methods, for Kalman filter. Furthermore, we proposed using HD map that allows the integration of local measurements and global measurements into a linear Kalman filter framework.

In the following, we will show how to formulate a linear Kalman filter, i.e., specifying the above parameters, to fuse the three types of inputs of GPS, monocular vision, and HD map, for vehicle localization. 

#### 2.3.1. Data-Driven Motion Model for State Transition

Unlike existing Kinematic model for vehicle localization, we proposed using a data-driven motion model for vehicle position prediction. The idea is to predict current vehicle position from its historic positions. In this model, the state of the vehicle is not represented with speed and acceleration. Instead, we use the current position $x_{k}$ and the *N* historic positions to formulate a trajectory consisting of *N +* 1 position as the current state as follows

(11) $$X_{k}={\left[\begin{array}{cccc} x_{k} & x_{k-1} & \ldots & x_{k-N} \end{array}\right]}^{T}\;$$

The position $x_{k}$ is a two-dimensional vector and takes the forms as

(12) $$x_{k}={\left[\begin{array}{cc} x_{k}^{x} & x_{k}^{y} \end{array}\right]}^{T}\;$$

By using the state transition matrix *F*, we can get the prediction of current state from its previous one as follows

(13) $$x_{k}=F_{k}x_{k-1}+Bu_{k}+w_{k},w_{k}∼N(0,\;Q_{k}),B=0\;$$

where $F_{k}$ is the state transition model which is applied to the previous state $x_{k-1}$; *B* is the control-input model which is applied to the control vector $u_{k}$; $w_{k}$ is the process noise which is assumed to be drawn from a zero mean multivariate normal distribution *N*, $Q_{k}$ is the covariance.

The Equation (13) is the basic prediction model that assumes the true state at time k is evolved from the state (speed, acceleration, etc.) at (*k* − 1). But in this paper, we use a data-driven motion model for vehicle position prediction. The parameters in the transition matrix *F* is computed from the training data. In this paper, the position of the vehicle is represented with *N* historic positions. The trajectory consists of a sequence of vehicle’s positions (two-dimensional vector) with the synchronized timestamp information. So, the position of the vehicle at state *k* can be expressed as follows

(14) $$x_{k}={\begin{array}{cccc} a_{1}x_{k-1}+ & a_{2}x_{k-2} & \cdots & +a_{N}x \end{array}}_{k-N}{}^{}=\sum_{i=1}^{N}a_{i}x_{k-i}\;$$

where $x_{k\;-\;N}$ is the position of vehicle at the state (*k − N*), $a_{N}$ is the weighting coefficient in $x_{k\;-\;N}$. Hence, we can generate linear constraint on the unknown $a={\left[a_{1}\;a_{2}\;\cdots \;a_{N}\right]}^{T}$ as follow

(15) $$\left[\begin{array}{cccc} x_{k-1} & x_{k-2} & \cdots & x_{k-N} \end{array}\right]{\left[\begin{array}{cccc} a_{1} & a_{2} & \cdots & a_{N} \end{array}\right]}^{T}=\left[\begin{array}{cccc} x_{k-1} & x_{k-2} & \cdots & x_{k-N} \end{array}\right]a\;$$

By stacking a number of states from the trajectory data, we can generate sufficient constraints to solve the efficiency $a\;={\left[a_{1}\;a_{2}\;\cdots \;a_{N}\right]}^{T}$.

Note that the number *N* is the order of the model. Higher *N* leads to better motion modeling capability yet with more computation complexity. For example, with *N* = 2, the model can incorporate dynamic velocities. With *N* = 3, the different accelerations can also be modeled. Compared to the complicated Kinematic model in the literature, the proposed model is data driven model. Hence, it requires no high-cost IMU to provide the acceleration and velocity data and is more flexible. Moreover, the model has the ability to cope with the constant speed, constant acceleration models, etc. For example, if *N* = 2, the model can cope with both the position and the speed. And the prediction matrix and the transition are taking the following form

(16) $$F=\left[\begin{array}{ccc} a_{1} & a_{2} & 0\\ 1 & 0 & 0\\ 0 & 1 & 0 \end{array}\right]\;$$

(17) $$X_{k}=FX_{k-1}=\left[\begin{array}{ccc} a_{1} & a_{2} & 0\\ 1 & 0 & 0\\ 0 & 1 & 0 \end{array}\right]\left[\begin{array}{c} x_{k-1}\\ x_{k-2}\\ x_{k-3} \end{array}\right]\;$$

Another issue for the prediction model in Kalman filter is the process noise problem. In this paper, the process noises are estimated from the model fitting errors. All the states from the training data are fitted with the computed efficiency and the fitting errors are computed to estimate the process errors. 

#### 2.3.2. Observations from GPS, Vision, and HD Map 

**Figure 3:**
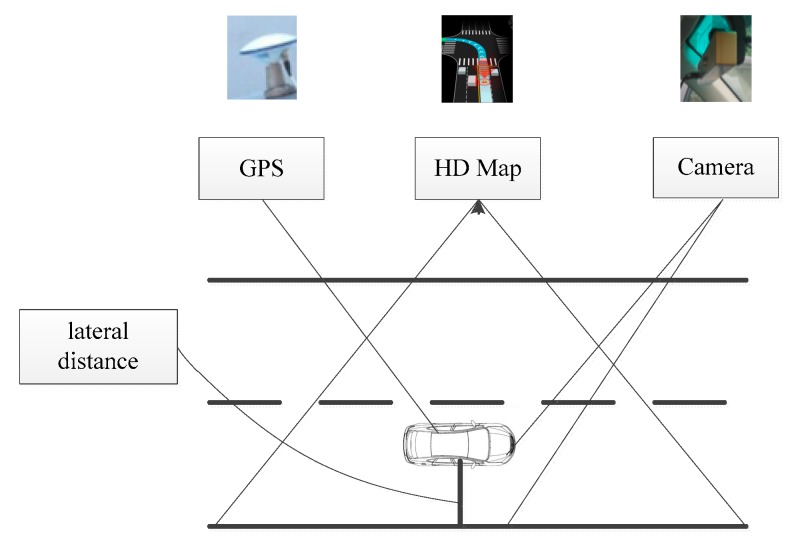
Lateral distance from monocular vision and GPS via HD map.

In this paper, we have two types of observations or measurements of the states as it shown in Figure 3. Through the two types of observations or measurements, we can compute the lateral distance via HD map. The first observation is directly from the raw coordinates from the GPS receiver. Thus, the observation matrix takes the forms as

(18) $$z_{g}=H_{g}X_{k}=\left[\begin{array}{cccc} 1 & 0 & \cdots & 0\\ 0 & 1 & \cdots & 0 \end{array}\right]\left[\begin{array}{c} x_{k}^{x}\\ x_{k}^{y}\\ \cdots \\ x_{k-n}^{x}\\ x_{k-n}^{y} \end{array}\right]\;$$

where $Z_{g}$ is the observation or measurement by GPS and $H_{g}$ is the observation model which maps the true state space into the observed space. By Equations (11) and (12) above, we can expand $X_{k}$ shown in Equation (18). We observe the values of GPS at the state *k*, so the $H_{g}=\left[\begin{array}{c} 1\;0\;\cdots \;0\\ 0\;1\;\cdots \;0 \end{array}\right]$.

The second observation is the lateral distance between the vehicle and the nearby lane. Particularly, the lateral distance is computed from the monocular vision. From HD map, we can also derive the geometric representation of the nearby lane denoted as $l={\left[\begin{array}{ccc} n_{x} & n_{y} & d \end{array}\right]}^{T}$ with $n_{x}^{2}+n_{y}^{2}=1$ from its high-accuracy GPS coordinates. As a result, we can compute the distance between the GPS coordinates from the GPS receiver and the lane by using the point-to-line formula from Equation (19) as follow. 

(19) $$d_{v}=l^{T}\left[\begin{array}{ccc} x_{k}^{x} & x_{k}^{y} & 1 \end{array}\right]=n_{x}x_{k}^{x}+n_{y}x_{k}^{y}+d\;$$

As a result, we can derive another observation as follow

(20) $$z_{v}=d_{v}-d\;$$

Hence, we can combine these two types of observations to formulate an integrated observation as follows

(21) $$z_{k}=\left[\begin{array}{c} Z_{g}\\ Z_{v} \end{array}\right]=H_{k}X_{k}=H_{k}\left[\begin{array}{c} x_{k}^{x}\\ x_{k}^{y}\\ \cdots \\ x_{k-n}^{x}\\ x_{k-n}^{y} \end{array}\right]\;$$

And the corresponding observation matrix takes the forms as

(22) $$H_{k}=\left[\begin{array}{c} H_{g}\\ H_{v} \end{array}\right]=\left[\begin{array}{cccc} 1 & 0 & \cdots & 0\\ 0 & 1 & \cdots & 0\\ n_{x} & n_{y} & \cdots & 0 \end{array}\right]\;$$

Note that, if we can detect both the left and the right lane lines from the camera, we can derive two observations from monocular vision, which are the lateral distances to the left and right lanes, respectively. In such a case, the observation matrix would have four columns rather than three in Equation (22). And the two-lane observation matrix takes the forms as

(23) $$H_{k}=\left[\begin{array}{c} H_{g}\\ H_{v} \end{array}\right]=\left[\begin{array}{l} \begin{array}{cccc} 1 & 0 & \cdots & 0\\ 0 & 1 & \cdots & 0\\ n_{xl} & n_{yl} & \cdots & 0 \end{array}\\ \begin{array}{cccc} n_{xr} & n_{yr} & \cdots & 0 \end{array} \end{array}\right]\;$$

In our method, we proposed using point-to-line distances rather than point-to-point distances as the observation, which is crucial to develop linear observation function for Kalman filter. Otherwise, we would need to deal with the non-linear observations (e.g., the point-to-point distances). Hence, more complicated non-linear Kalman filter, such as Extended Kalman filter (EKF) or Unscented Kalman filter (UKF) should be applied to cope with such non-linear observations. 

## 3. Experimental Results and Discussions

This section may be divided by subheadings. It should provide a concise and precise description of the experimental results, their interpretation as well as the experimental conclusions that can be drawn.

In this paper, we used different test data (simulation data and real data) to validate the proposed algorithm. A driving simulator was built to collect simulation data, where different driving conditions can be configures. In addition, a real experiment was performed on the route along Youyi road in Wuhan City, China covering both urban roads and roads on campus.

### 3.1. Test Results with Simulation Data

**Figure 4:**
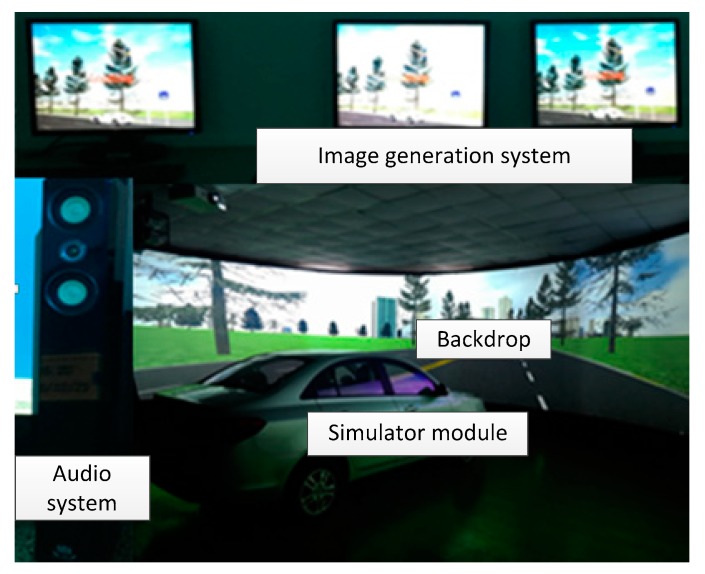
Driving simulator to generate simulation data.

As shown in Figure 4. The driving simulation is composed of audio system, simulator module, backdrop and image generation system. Simulator module is a Citroen vehicle. Audio system is composed of four audios. Visual system is mainly used to generate and display in the virtual traffic scene, its components include the display system, projection system and graphical simulation systems. With these equipment, the driving simulator can simulate real driving experiment very well. We can get the data of camera and GPS like a real vehicle. It can provide a front—view picture with 800 × 600 pixel and the coordinates of vehicle.

**Figure 5:**
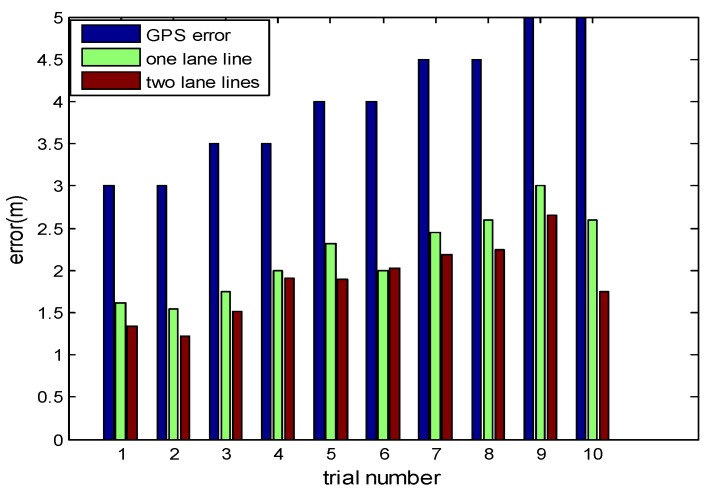
The errors of vehicle localization.

With the driving simulator, we can set different driving environment and different data error we need. We can get all the environment data in the different environment, like the coordinates of lane lines etc. As it shown in the Table 1, we set the error of GPS from 3 to 5 m and the error of lateral distance from lane line to the vehicle by monocular vision to 0.1 m. As it shown in Figure 5, if two lanes were used for reference, the localization errors can drop by 10%–30%.

### 3.2. Test Results with Real Data

**Figure 6:**
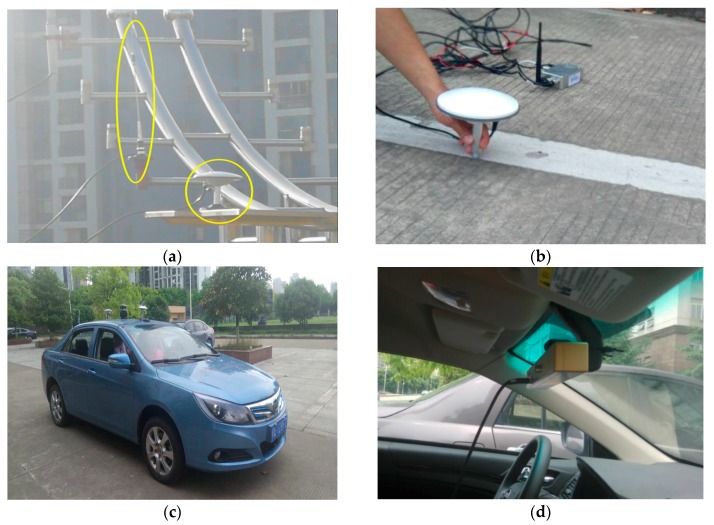
Data acquisition system: (**a**) RTK base station, (**b**) lane coordinate acquisition, (**c**) test vehicle, (**d**) vision system.

In the real test, we used a prototype intelligent vehicle developed by WUT for real data collection. As shown in Figure 6, the data collection system was composed of the RTK (Real-time kinematic) base station, the lane coordinate collect system, the experiment vehicle which could receive GPS and RTK signals, and the vision system. Based on the RTK station built by ourselves, we can get the coordinate information in a precision of 1 cm. So we used it to collect the coordinates of lanes and vehicle. The data collected by the GPS and monocular vision were used as the observation and those collected by RTK as the ground truth. 

As it shown in Figure 6c, it is the BYD electric car modified by us. In the Figure 6d, the cars’ interior front height is about one meter from the ground. The vision system could provide the front image include lane lines, the image is 800 × 600 pixel. 

**Figure 7:**
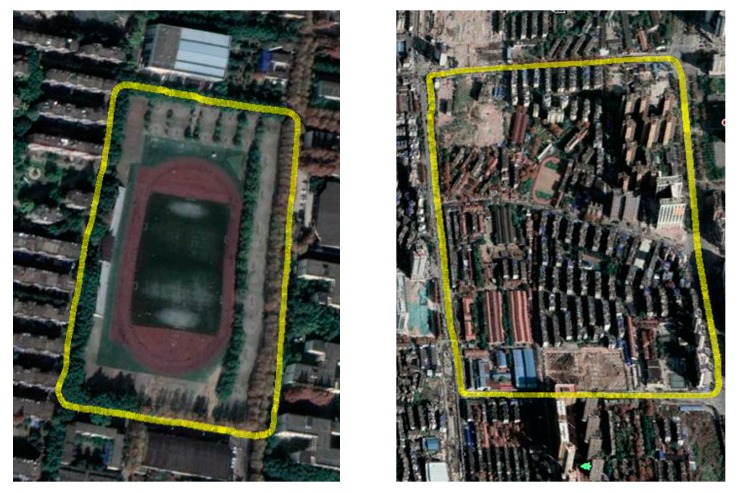
Test route around Youyi road.

The experimental route is shown in Figure 7, it has two kinds of driving environment, include urban road and school road. The city roads section is along Youyi road in Wuhan City, China and the school road is in the Wuhan University of Technology in Wuhan City, China. They include different environments to test the method proposed in this paper.

**Figure 8:**
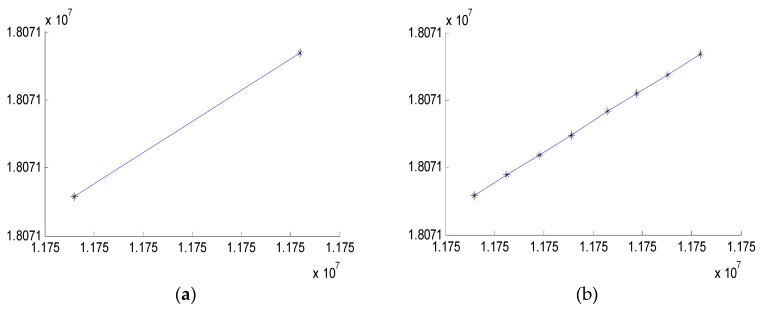
The coordinate of lane lanes in UTM. (**a**) full lane line; (**b**) dotted lane line.

After choosing the experimental route, we collect the coordinates of lane lines around the road as shown in Figure 7. In order to obtain accurate linear expression of lane line, we choose these points (starting point and ending point on the lane) on each lane line. Then the points in latitude and longitude coordinates would transform into geodetic coordinates by UTM as shown in Figure 8, through the several points we would compute the linear expression of lanes.

Table 2 shows the extracted lines from the 12 test images, according to the proposed approach. Based on the info above, calibrated camera parameters and vanishing points are used to calculate D_l_, D_r_. D_l_ is the distance to the left lane and D_r_ is the distance to the right lane.

**Figure 9:**
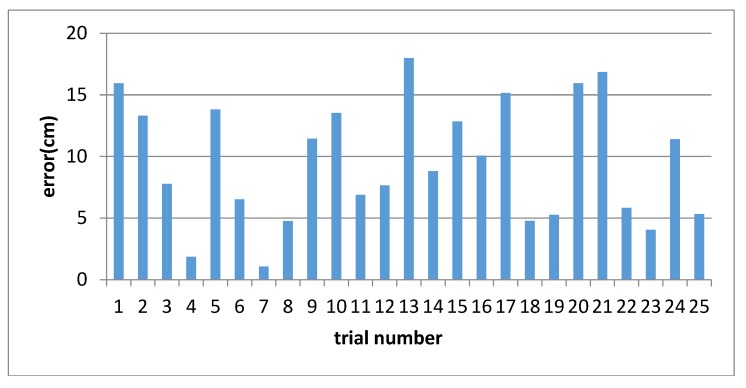
The lateral error of vehicle to the lanes.

As shown in Figure 9, we can observe that the most errors are below 15 cm and its average error is 9.55 cm. The results showed that the methods mentioned had a positive effect. The data could be used for Kalman filter below.

**Figure 10:**
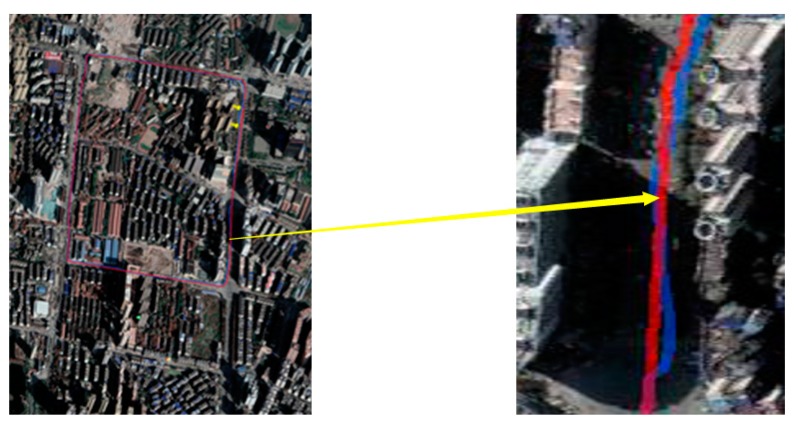

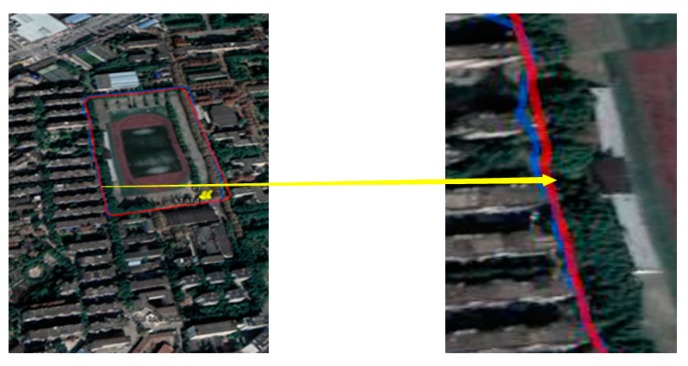
Coordinate of lanes on GIS (Geo-Information System) map.

When we driving in the test route, the latitude and longitude coordinates wound be collected by the GPS and RTK. The blue and red curves in Figure 10 show the route of vehicle driving in different environment by different sensors. The blue curve shows the route acquisition through GPS and the red curve is the route acquisition by RTK. The red curve is the ground truth in this paper and the blue curve is the observation value in Kalman filter.

**Figure 11:**
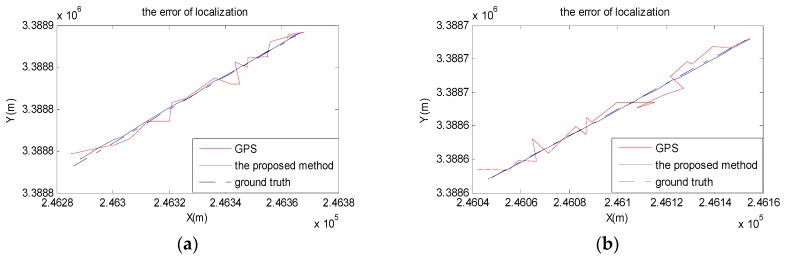
The results of localization. (**a**) urban road; (**b**) school road.

From the localization results shown in Figure 11, the red curve is for GPS coordinates, while the black curve is for RTK coordinates, which is the ground truth in the experiment. The blue curve is for the results by using our method proposed in this paper. We can see that the curve from the proposed method not only become closer to the ground truth, but also become smoother.

**Figure 12:**
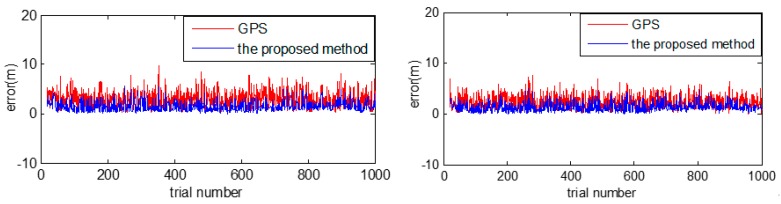
Localization errors from the proposed method.

As shown in Figure 12, it is a numerical comparison on displacement errors, among noised GPS measurements (red), vehicle position after the proposed method (blue). The X axis represents trial number and the Y axis represents the error (mm) close to the ground truth. And the blue curve is closer to ground truth than the red curve, which implies that Kalman filter based localization improves GPS based localization. 

**Figure 13:**
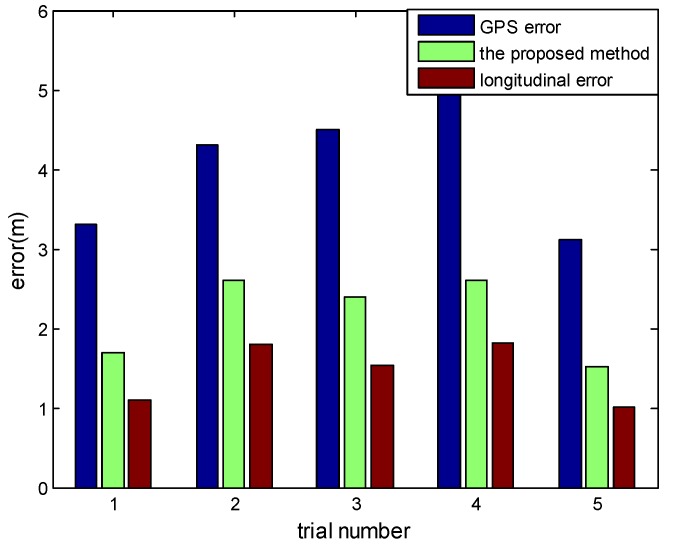
Errors of vehicle localization.

The lateral error can be got from the monocular vision in an accuracy way, so the longitudinal error could response the result of vehicle localization to a certain degree. As it shown in Table 3 and Figure 13, we have counted the errors of positioning error and longitudinal error in several times experiment. In our method, we can reduce the error to less than half or even one-third of the original GPS positioning errors by using low cost sensors with HD map support, which numerically proves that Kalman filter based localization helps to improve the performance of vehicle localization.

The proposed method can be easily integrated into existing localization methods to further enhance vehicle localization results, because it utilizes novel constraints on the vehicle’s locations. In this part, we chose three typical localization methods in Refs. [15,16,28] on the same test route. Among them, the ORB-SLAM in [28] is a well-acknowledged method. And the methods in [30,31] are two state-of-the-art ones. For each localization method, we compute the mean positioning errors. For comparison, we also integrated the proposed method into existing one and computed the localization results. The mean positioning errors were also computed. As a result, we can evidently compare the localization results with and without integrating the proposed method. The results are illustrated in Table 4. It can be observed in Table 4, although the localization errors are different for the three different methods, the integration of the proposed method can reduce the positioning errors by 23.4%–33.3% for all these three different methods. 

## 4. Conclusions

Vehicle localization is an important issue for intelligent vehicles. In this paper, we proposed a low-cost and accurate solution to vehicle localization. In this solution, we proposed using Kalman filter to fuse GPS, monocular vision and HD map to enhance localization precision. In the Kalman filter, both raw GPS and lateral distance from monocular vision were used as the observations. In particular, the HD map was used to correlate GPS coordinates and lateral distance to generate point-to-line distance as linear constraints on the states. Moreover, we proposed using a data-driven motion model, compared to Kinematic model in existing methods, for more adaptive and flexible state prediction and transition. The data-driven model is trained from vehicle’s historic trajectories and requires no acceleration or velocity inputs, which therefore requires no high-cost equipment. The proposed method was tested with both simulation and real data, collected by a simulator and a prototype intelligent vehicle along the Youyi road in Wuhan, China, respectively. Experiment results show that the localization errors from the proposed method are about 30%–50% of the raw GPS errors. Compared to one lane, the localization errors can drop by 10%–30% if two lanes were used for reference. Experimental results also demonstrate that the proposed method can be easily integrated into existing methods to further enhance localization results. The proposed method suggests a novel solution to intelligent vehicle localization. 

## Figures and Tables

**Table 1:** Setting of data errors in the simulator.

Data Type	Mean (m)	SD (m)
Ground truth data	0	0
Raw GPS coordinates	3–5	3
Lateral distance from lane line	0.1	0.05

**Table 2:** Result of self-localization.

Trial Number	D_r_ (mm)	D_l_ (mm)
1-1	1800	1800
1-2	1877.2	1784.5
1-3	1829.4	1797.7
1-4	2199.2	1486.2
1-5	2531.8	1140.2
1-6	2788.4	893.32
1-7	2789.5	891.1
1-8	3285.1	485.28
1-9	2763.9	868.87
1-10	2153.5	1494.7
1-11	1802.9	1876.8
1-12	1780.8	1881.7

**Table 3:** Localization errors from different values of GPS errors.

Positioning Error of GPS (m)	Positioning Error Mean (m)	Positioning Error SD (m)	Longitudinal Error Mean (m)	Longitudinal Error SD (m)
4.33	2.79	2.55	1.83	1.79
3.75	1.27	1.18	0.91	0.67
4.04	1.88	1.94	1.43	1.18
3.12	1.51	1.39	0.98	1.03

**Table 4:** Comparison of localization results with/without integration of the proposed method.

Methods	Mean Localization Errors (m)	Error Reduction after Integration (%)
Method in [16]	11.12	33.3%
Method in [16] + Proposed method	7.42	
Method in [28]	0.54	31.5%
Method in [28] + Proposed method	0.37	
Method in [15]	2.91	23.4%
Method in [15] + Proposed method	2.23	
